# The effect of outdoor sports education on university students’ employment anxiety: the mediating role of fear of failure and self-management

**DOI:** 10.1186/s40359-026-04498-2

**Published:** 2026-04-02

**Authors:** Hailong Chang, Chao Chen, Shaolong Liu, Danyang Wang, Chuang Yan, Lei Wang, Feng Bao, Minghao Yan, Yinqiao Guo, Haijiang Shen

**Affiliations:** 1 Department of Physical Education, Tangshan Normal University, Tangshan City, Hebei Province China; 2 Department of Physical Education, Shijiazhuang Institute Of Technology, Shijiazhuang City, Hebei Province China; 3 Department of Physical Education, Hebei Software Institute, Baoding city, Hebei Province China; 4 Department of Physical Education, Hebei Normal University, Shijiazhuang city, Hebei Province China

**Keywords:** Outdoor sports education, Employment anxiety, Mediating role, Self-management, Fear of failure

## Abstract

**Background:**

University students are at a critical stage of professional socialization, where intense competition in the job market exposes them to considerable employment challenges and pressure. These factors often lead to negative expectations regarding future career prospects and contribute to the development of employment anxiety. This study aimed to investigate the relationship between outdoor sports education and employment anxiety using a combined longitudinal and cross-sectional design. Specifically, the research examined both the long-term effects and immediate associations of this relationship, as well as the potential mediating roles of self-management and fear of failure.

**Methods:**

A total of eight classes were selected and randomly allocated to either an intervention group (four classes) or a control group (four classes). The intervention group participated in a structured outdoor sports education program, while the control group engaged in conventional physical activities (e.g., basketball, aerobics). Employment anxiety was measured using the Future Employment Anxiety Scale, self-management was assessed with the Self-Management Questionnaire, and fear of failure was measured using the Performance Failure Appraisal Inventory. Data were analyzed using independent-samples t-tests, descriptive statistics, correlation analysis, and mediation analysis. Mediation effects were examined through Baron and Kenny’s causal steps approach, supplemented by 5,000 bootstrap iterations to assess the statistical significance of indirect effects.

**Results:**

Following the intervention, students in the intervention group showed significantly lower levels of anxiety in the domains of Personal Ability, Career Replaceability, and Social Relations compared to the control group (*p* < .05). Conversely, they exhibited significantly higher scores across all four dimensions of self-management: Behavior Management, Emotion Management, Time Management, and Cognition Management (*p* < .05). Furthermore, the intervention group reported significantly lower scores on three dimensions of fear of failure—Fear of Experiencing Embarrassment, Fear of Devaluation by Others, and Fear of Others Losing Interest (*p* < .05). Regarding effect decomposition, outdoor sports education accounted for 28.1% of the total effect, reflecting a significant direct predictive role on employment anxiety. The indirect effect mediated by self-management contributed 26.3%, while that mediated by fear of failure explained 33.3% of the total effect. Additionally, the chain-mediated pathway involving both self-management and fear of failure accounted for 12.3% of the total effect.

**Conclusions:**

Outdoor sports education was associated with lower employment anxiety, enhanced self-management competencies, and reduced fear of failure among university students. Both self-management and fear of failure function as independent mediating factors in the link between outdoor sports education and employment anxiety. Furthermore, these two variables operate sequentially in a chain-mediation pathway, jointly accounting for a meaningful proportion of the overall effect.

**Trial registration:**

ISRCTN Registry (ISRCTN14022322). Registered on 30 March 2026. Retrospectively registered.

## Introduction

 The expansion of higher education has led to a consistent increase in university enrollments, resulting in a growing number of graduates annually. In China, for instance, the graduating cohort of 2025 is projected to reach a historic high of 12.22 million. While the overall employment landscape remains relatively stable, graduates encounter distinct challenges during their job search. On one hand, emerging industries such as artificial intelligence, new energy, and biomedicine exhibit strong demand for specialized talent. On the other hand, employment opportunities in several traditional sectors have diminished. Compounding this situation is a pronounced skills mismatch, as many graduates lack the practical competencies required to meet employers’ immediate needs [1]. According to the China National Mental Health Development Report, employment anxiety has emerged as a significant mental health concern among university graduates [2]. University students are at a pivotal stage of professional socialization, where intense labor market competition exposes them to substantial employment pressure and challenges, often fostering pessimistic outlooks regarding future career prospects and contributing to the onset of employment anxiety [3]. Such anxiety not only undermines the psychological well-being of students but also impairs the effectiveness of institutional career services and may adversely influence their eventual employment outcomes [4].

Employment anxiety is defined as a state of emotional tension and unease experienced by college students when they perceive themselves as unlikely to attain their desired employment outcomes [5]. Recent research has found that employment anxiety represents a prevalent psychological issue in this population, with many students reporting significant levels of anxiety characterized by heightened tension and other negative psychological symptoms [6]. While mild anxiety may serve an adaptive function by mobilizing personal resources and enhancing motivation to confront career-related challenges, excessive or chronic anxiety—beyond a critical threshold—can undermine social functioning, impede effective problem-solving, disrupt daily activities, and potentially escalate into clinical anxiety disorders or maladaptive coping behaviors, thereby increasing the risk of academic and occupational setbacks [7]. Given its direct implications for students’ mental well-being, career decision-making, and employment prospects, it is imperative to investigate the underlying mechanisms of employment anxiety and formulate evidence-based intervention strategies.

Fear of failure is conceptualized as a negative affective response that occurs when individuals anticipate being unable to attain a desired goal in achievement-related contexts [8]. In such situations, individuals typically engage in a cognitive appraisal of both task difficulty and their own capabilities. If they perceive themselves as competent to succeed, positive emotions such as satisfaction and excitement are likely to follow [9]. Conversely, when they judge their ability as insufficient or anticipate a high probability of failure, the task is perceived as threatening, evoking anxiety and apprehension. In the contemporary context—characterized by both intense competition and abundant opportunities—university students are particularly susceptible to heightened fear of failure [10]. This fear can undermine psychological resilience, elevate general anxiety levels, and, when severe, contribute to negative emotional states including anxiety and depression [11]. Consequently, fear of failure represents a key psychological factor that not only exacerbates anxiety among students but also amplifies employment-related distress.

Self-management encompasses a set of regulatory processes through which individuals engage in self-awareness, self-evaluation, self-discipline, and self-motivation to promote personal development [12]. Grounded in agency theory—which posits that individuals possess the capacity for self-regulation and proactive adaptation—self-management aligns with educational philosophies that emphasize learner autonomy [13]. This perspective is further reinforced by theories of holistic education, which hold that fostering self-discipline and adaptability is central to helping individuals realize their potential and engage productively with the world around them [14].

From a human development perspective, nurturing self-management skills is regarded as integral to holistic personal growth and essential for meeting the developmental needs of university students [15]. Outdoor sports education is an experiential pedagogical approach that uses outdoor physical activities as a medium to integrate multidisciplinary knowledge and skills, involving various stakeholders with the aim of enhancing participants’ physical and psychological well-being [16]. Its curriculum should incorporate multi-level and cross-disciplinary content, extending beyond technical outdoor skills to include elements of natural science, cultural literacy, social responsibility, and teamwork, thereby enriching its educational value [17]. Through deliberately designed, challenging, and goal-oriented activities, outdoor sports education fosters constructive psychological attributes and proactive life attitudes. Empirical evidence suggests that its distinctive pedagogical format can effectively mitigate negative emotional states such as anxiety and depression among participants [18].

In summary, against a backdrop of rapidly evolving labor markets and mounting employment pressure, university students are confronting increasingly severe levels of employment anxiety. Fostering psychological resilience during academic training and maintaining well-being throughout the school-to-work transition have thus become imperative. Fear of failure, a significant contributor to anxiety and a key concern for student mental health, underscores the critical need to cultivate constructive orientations toward setbacks. Self-management encompassing self-organization and self-control capacities enhances cognitive flexibility in career adaptation. Individuals with well-developed self-management skills are better equipped to direct attention toward positive cues, form adaptive attributions, and experience more favorable emotional responses in employment contexts [19]. Notably, outdoor sports education has been shown to promote positive psychological traits in students. The inherently challenging nature of its activities is conceptually linked to fear of failure, while its diverse instructional settings and demands inherently call for proficient self-management [20]. Grounded in this theoretical framework, the present study pursued three primary objectives: (1) to examine the effect of a 16-week outdoor sports education program on university students’ employment anxiety (2) to investigate the mediating roles of self-management and fear of failure in the relationship between outdoor sports education and employment anxiety; and (3) to explore the chain mediation effect of self-management and fear of failure in this association.

## Methods

### Design

This study employed a combined longitudinal and cross-sectional design to examine both the immediate associations and sustained effects of outdoor sports education on employment anxiety. In the longitudinal phase, an extended experimental intervention was conducted to systematically assess the overall impact of outdoor sports education on university students’ employment anxiety, self-management, and fear of failure. Based on the empirical findings, a theoretical mediation model was developed, positioning self-management and fear of failure as key mediators to elucidate the underlying mechanisms and boundary conditions through which outdoor sports education influences employment anxiety. This study was registered with the ISRCTN Registry (ISRCTN14022322) and received ethical approval from the Institutional Review Board of the Tangshan Normal University (TSTC2025-8-10).

### Participants and procedure

This study recruited undergraduate students enrolled in compulsory physical education courses at Tangshan Normal University. To ensure adequate statistical power to detect the hypothesized effects, an a priori power analysis was conducted using G*Power software (Version 3.1). For the primary analysis—independent-samples t-tests comparing post-intervention outcomes between groups—we specified a significance level (α) of 0.05, a desired power (1 − β) of 0.80, and anticipated a medium effect size (Cohen’s d = 0.5) based on prior literature in similar educational interventions. This analysis indicated that a minimum of 64 participants per group would be necessary. Following screening for injuries, mental health concerns, or other conditions that might preclude safe participation, seven students were excluded. The final sample therefore comprised 310 participants, with a mean age of 19.23 years (SD = 0.67).

A cluster randomized design was employed, with class as the unit of randomization to avoid contamination between conditions. Eight intact classes were randomly allocated to either the intervention group (four classes, 157 participants) or the control group (four classes, 153 participants). Randomization was performed by an independent researcher using a computer-generated random number sequence (random.org). To ensure allocation concealment, class assignments were placed in sealed, opaque envelopes and opened only after baseline data collection. Baseline equivalence between groups was confirmed through independent-samples t-tests on all outcome measures (all *p* > .05; see Table 2). The final sample size substantially exceeds the minimum requirement of 64 participants per group, thereby providing sufficient statistical power for the subsequent analyses, even accounting for potential attrition and the cluster-randomized design. In all correlation and mediation analyses, age was treated as a continuous variable to maximize statistical power and avoid potential bias associated with arbitrary categorization.

**Fig. 1:**
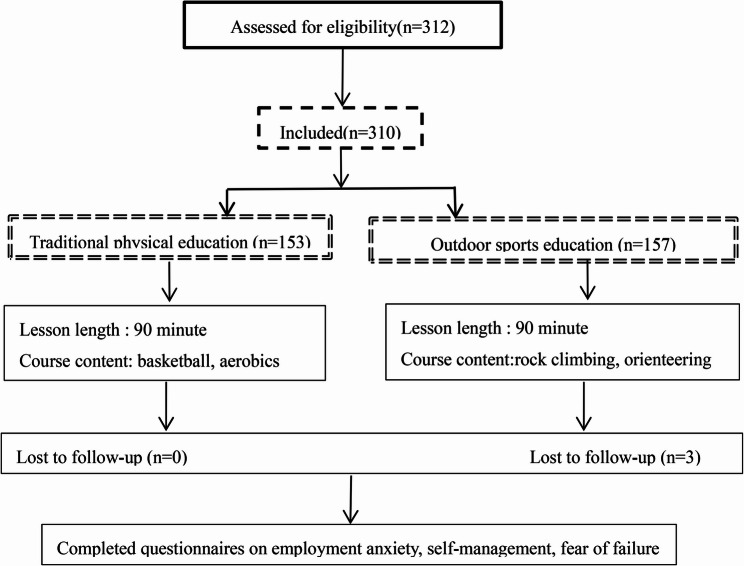
Research procedure

### Intervention

The 16-week outdoor sports education intervention program was designed based on experiential learning theory and the outdoor education framework of Priest and Gass, emphasizing: (a) direct experience in natural environments, (b) progressively challenging tasks, (c) group-based problem-solving, and (d) structured reflection (see Table 1). The intervention comprised four progressive phases. During Weeks 1–3 (Foundation), students engaged in trust exercises, basic navigation skills training, and low ropes elements, with low intensity focused on building psychological safety and group cohesion. Weeks 4–8 (Individual challenge) involved indoor rock climbing at moderate to high intensity, where students practiced basic techniques, belaying, goal-setting, and managing fear of heights. Weeks 9–12 (Group cooperation) featured orienteering tasks at moderate intensity, including small-group navigation and time-pressured multi-point challenges requiring collaborative decision-making. Finally, Weeks 13–16 (Application) comprised multi-stage tasks, group problem-solving scenarios (e.g., river crossing simulation, shelter building), and a student-planned mini-expedition, with variable intensity emphasizing autonomous decision-making and teamwork.

In contrast, students in the control group participated in conventional physical education classes as prescribed by the university curriculum, including basketball or aerobics, with their respective classes. The basketball curriculum consisted of skill drills (e.g., dribbling, shooting, passing) and small-sided games. The aerobics curriculum involved choreographed routines set to music, focusing on cardiovascular fitness, coordination, and flexibility. The intervention lasted for 16 weeks, with one 90-minute session per week. Importantly, all control group sessions were conducted in outdoor settings and explicitly excluded outdoor adventure elements, natural environment exposure, and structured psychological debriefing. This design ensured that the control condition served as a valid baseline for isolating the unique effects of the outdoor sports education intervention.

**Table 1:** Summary of the 16-week outdoor sports education intervention

Period	Exercise focus	Exercises/Activities	Intensity
Week 1–3	Foundation	Trust exercises, navigation basics, low ropes	Low
Week 4–8	Individual challenge	Rock climbing	Moderate-high
Week 9–12	Group cooperation	Orienteering	Moderate
Week 13–16	Application	Multi-stage tasks, expedition	Variable

### Measures

The Future Employment Anxiety Scale, developed by Chen Wang et al. [20], was constructed through qualitative interviews and iterative item generation. Its correlated four-factor structure—comprising Personal Ability, Knowledge Application, Career Replaceability, and Social Relations—was established and validated via exploratory and confirmatory factor analyses. Responses are recorded on a 5-point Likert scale (1 = strongly disagree, 5 = strongly agree), with higher total scores reflecting greater levels of employment anxiety. In the present study, the scale demonstrated high internal consistency, with Cronbach’s α coefficients of 0.965, 0.971, 0.955, and 0.965 for the respective subscales. Furthermore, a confirmatory factor analysis (CFA) was conducted to re-examine its construct validity within our specific sample. The correlated four-factor model demonstrated an acceptable fit to the data: χ²/df = 2.86, CFI = 0.94, TLI = 0.92, RMSEA = 0.06, SRMR = 0.04. Factor loadings for all items were statistically significant and ranged from 0.68 to 0.91. The composite reliability (CR) for the four factors ranged from 0.87 to 0.93, exceeding the recommended threshold of 0.70, and the average variance extracted (AVE) ranged from 0.62 to 0.71, surpassing the 0.50 benchmark, thus supporting good internal consistency and convergent validity.

The Self-Management Questionnaire compiled by Zhang Guoli et al. [21] was administered to assess self-regulation capacities. The instrument contains 42 items distributed across four dimensions: behavior management, emotion management, time management, and cognition management. Items are rated on a 5-point Likert scale ranging from 1 (completely inconsistent) to 5 (completely consistent), with 12 items reverse-scored. Higher total scores indicate stronger self-management competencies. In this sample, the full scale exhibited excellent internal consistency (Cronbach’s α = 0.941). Subscale reliability coefficients were as follows: behavior management = 0.837, emotion management = 0.797, cognition management = 0.816, and time management = 0.826. Additionally, a CFA was performed to assess the structural validity of this instrument in our sample. The hypothesized four-dimensional structure yielded a satisfactory model fit: χ²/df = 2.91, CFI = 0.92, TLI = 0.91, RMSEA = 0.06, SRMR = 0.05. All factor loadings were significant (range: 0.64–0.88). The CR values for the four dimensions ranged from 0.84 to 0.90, and the AVE values ranged from 0.58 to 0.68, indicating adequate convergent validity.

Fear of failure was measured using the Chinese adaptation of the Performance Failure Appraisal Inventory, originally developed by Conroy et al. and adapted by Zhuo Guoxiong and Lu Junhong [22]. The scale consists of 18 items (e.g., “When I fail, I blame myself for lacking talent”) organized into four dimensions: fear of experiencing embarrassment, fear of devaluation by others, fear of others losing interest, and fear of disappointing important others. Responses are given on a 5-point Likert scale from 1 (strongly disagree) to 5 (strongly agree), with higher total scores representing stronger fear of failure. The scale showed good internal consistency in this study (Cronbach’s α = 0.79). A CFA was also conducted to test the four-factor structure within our dataset. The model exhibited a good fit to the data: χ²/df = 2.73, CFI = 0.95, TLI = 0.93, RMSEA = 0.05, SRMR = 0.04. Item factor loadings ranged from 0.71 to 0.89. The CR values for the four subscales (Fear of Experiencing Embarrassment, Fear of Devaluation by Others, Fear of Others Losing Interest, Fear of Disappointing Important Others) ranged from 0.86 to 0.91, and the AVE values ranged from 0.60 to 0.69, confirming the scale’s robust psychometric properties in the current sample.

### Statistical analysis

All statistical analyses were conducted using SPSS Statistics (Version 26.0, IBM Corp). Prior to hypothesis testing, key statistical assumptions were examined. Normality was assessed through skewness and kurtosis values, with all variables falling within acceptable ranges. Univariate outliers were screened using standardized z-scores, and multivariate outliers were assessed using Mahalanobis distance (*p* < .001); no extreme cases requiring exclusion were identified. Linearity was confirmed through visual inspection of scatterplots. Given that randomization was conducted at the class level, intra-class correlation coefficients (ICCs) were calculated for the primary outcome variables. ICC values ranged from 0.02 to 0.05, all below the conventional threshold of 0.10, indicating negligible clustering effects and justifying the use of single-level analyses. Multicollinearity diagnostics revealed that all predictor variables exhibited VIF values below 3.0 and tolerance values above 0.40, well within the recommended thresholds. To justify the adequacy of the sample size, both a priori and post hoc power analyses were conducted using G*Power. The final sample (*N* = 312) substantially exceeded this requirement. Post hoc power analysis based on the observed effect sizes revealed that statistical power for the main comparisons all exceeded the recommended 0.80 threshold.

Independent-samples t-tests were used to compare the intervention and control groups on fear of failure, self-management, and employment anxiety. Pearson’s correlation coefficients were calculated to examine bivariate associations among the study variables, with significance levels set at *p* < .05 and *p* < .01. To investigate the mediating roles of fear of failure and self-management in the relationship between outdoor sports education and employment anxiety, Baron and Kenny’s three-step mediation procedure was employed. Given that preliminary analyses revealed significant gender differences in employment anxiety and fear of failure (see Table 2), gender was included as a covariate in all subsequent regression and mediation analyses to control for its potential confounding effects. In addition to testing the direct relationships via regression equations, the bootstrapping method with 5,000 resamples was applied to evaluate the reliability of the indirect effects. Bias-corrected 95% confidence intervals (CIs) were computed; an effect was considered statistically significant if its 95% CI did not include zero. Missing data were addressed through multiple imputation, which involved generating several complete datasets, analyzing each independently, and then pooling the estimates to minimize bias.

**Table 2:** Descriptive statistics of outcome variables by gender and group

Demographic		Employment anxiety	Self-management	Fear of failure
Gender	Male	4.11 ± 0.51	3.28 ± 0.28	4.29±0.037
	Female	4.53 ± 0.39	3.32 ± 0.31	4.45 ± 0.55
	P	< 0.05	> 0.05	< 0.05
Group	Intervention group	4.30 ± 0.50	3.29 ± 0.29	4.38 ± 0.52
	Control group	4.34 ± 0.34	3.21 ± 0.44	4.36 ± 0.36
	P	> 0.05	> 0.05	> 0.05

Note: Age was treated as a continuous variable in subsequent analyses (M = 19.23, SD = 0.67). No significant age effects were found on any study variables

### Result

#### Overview of analyses

**Fig. 2:**
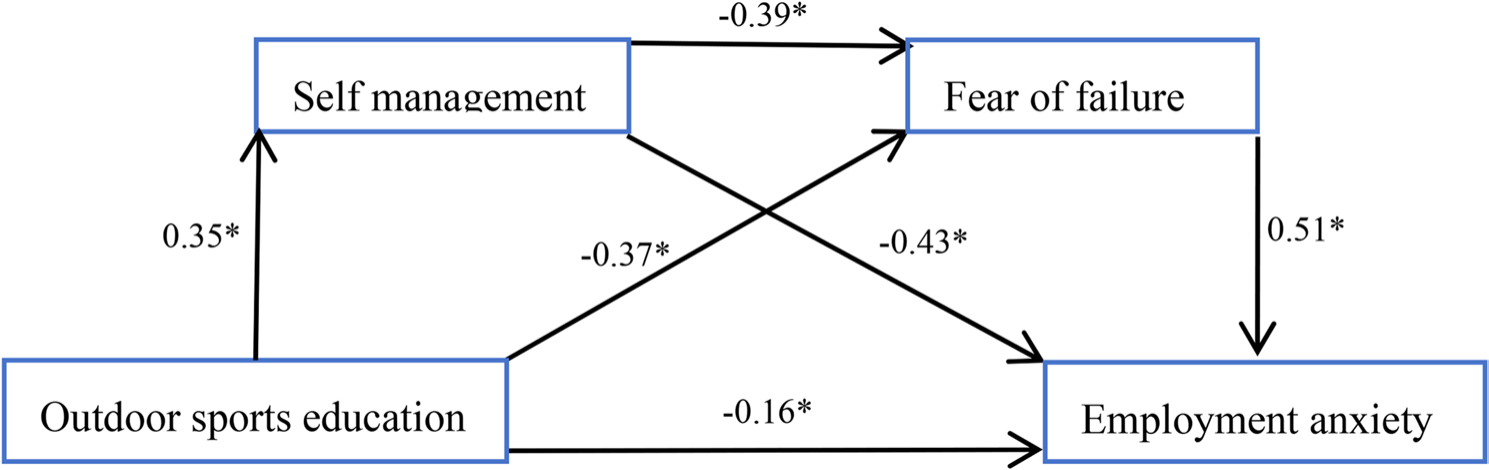
Mediation model showing role of fear of failure and self management on relationship between outdoor sports education and employment anxiety. Values shown are standardized coefficients*p < .05

The results are presented in two sections. First, intervention effects were examined by comparing the intervention and control groups on employment anxiety, self-management, and fear of failure using ANCOVA (Tables 3, 4 and 5). Second, to investigate the relationships among the key variables and test the proposed mediation model, data from both groups were combined (*N* = 307) for correlation analysis (Table 6) and structural equation modeling (Table 7; Fig. 2).

**Table 3:** ANCOVA results for group differences in employment anxiety

Personal Ability		Pre-test	Post-test	F	*P*	η²*p*
	Intervention	4.28 ± 0.48	3.55 ± 0.37	8.42	0.002	0.12
	Control	4.32 ± 0.35	3.93 ± 0.21			
Knowledge Application	Intervention	4.25 ± 0.45	3.61 ± 0.23	2.13	0.146	0.03
	Control	4.29 ± 0.38	3.76 ± 0.28			
Career Replaceability	Intervention	4.33 ± 0.52	3.70 ± 0.31	7.86	0.005	0.11
	Control	4.37 ± 0.40	4.19 ± 0.35			
Social Relations	Intervention	4.22 ± 0.47	3.32 ± 0.24	9.14	0.003	0.13
	Control	4.26 ± 0.39	3.79 ± 0.30			
	Intervention	4.30 ± 0.50	3.55 ± 0.24	8.97	0.003	0.13
Total Score	Control	4.34 ± 0.34	3.92 ± 0.23			

Note: Adjusted means are estimated with pre-test scores and gender held constant at their means. F-values and effect sizes are from ANCOVA models including both covariates

### Descriptive statistics of outcome of participation

Table 2 presents the comparative analysis of key variables across demographic groups. Preliminary analyses revealed no significant gender differences in self-management (*p* > .05), although female students reported higher levels of employment anxiety and fear of failure than males (*p* < .05). Age, treated as a continuous variable, showed no significant correlations with any of the main study variables (all *p* > .05). Importantly, no significant pre-intervention differences were found between the intervention and control groups on any measure (all *p* > .05), confirming successful random assignment.

### Differences in employment anxiety after the experiment

After controlling for pre-test scores and gender, ANCOVA revealed significant intervention effects on three employment anxiety dimensions (Table 3). The intervention group reported significantly lower scores than controls on Personal Ability (η²*p* = .12), Career Replaceability (η²*p* = .11), and Social Relations (η²*p* = .13), all with medium-to-large effects. Knowledge Application showed no significant group difference (η²*p* = .03). Total employment anxiety was significantly lower in the intervention group (η²*p* = .13, Cohen’s d = 0.74). These findings suggest that outdoor sports education is associated with reduced employment anxiety among university students.

### Differences in self-management after the experiment

After controlling for pre-test scores and gender, ANCOVA revealed significant intervention effects on all four self-management dimensions (Table 4). The intervention group reported significantly higher scores than controls on Behavior Management (η²*p* = .14), Emotion Management (η²*p* = .12), Time Management (η²*p* = .11), and Cognition Management (η²*p* = .12), with medium-to-large effects. Total self-management was significantly higher in the intervention group (η²*p* = .13, Cohen’s d = 0.76). These findings suggest that participation in outdoor sports education was linked to enhanced self-management competencies.

**Table 4:** ANCOVA results for group differences in self-management

Behavior management		Pre-test	Post-test	F	*P*	η²*p*
	Intervention	3.31 ± 0.30	3.95 ± 0.26	9.23	0.002	0.14
	Control	3.23 ± 0.45	3.32 ± 0.43			
Emotion management	Intervention	3.28 ± 0.28	4.27 ± 0.28	8.76	0.003	0.12
	Control	3.20 ± 0.43	3.69 ± 0.35			
Time management	Intervention	3.27 ± 0.27	4.08 ± 0.32	7.94	0.005	0.11
	Control	3.19 ± 0.42	3.61 ± 0.30			
Cognition management	Intervention	3.30 ± 0.29	4.03 ± 0.37	8.21	0.004	0.12
	Control	3.22 ± 0.44	3.57 ± 0.37			
	Intervention	3.29 ± 0.29	4.08 ± 0.26	9.02	0.003	0.13
Total Score	Control	3.21 ± 0.44	3.55 ± 0.31			

### Differences in fear of failure after the experiment

As shown in Table 5, after controlling for pre-test scores and gender, ANCOVA revealed that students in the intervention group reported significantly lower scores on three fear of failure dimensions: Fear of Experiencing Embarrassment (η²*p* = .12), Fear of Devaluation by Others (η²*p* = .13), and Fear of Others Losing Interest (η²*p* = .11), all with medium-to-large effects. Fear of Disappointing Important Others showed no significant group difference (η²*p* = .03). Total fear of failure was significantly lower in the intervention group (η²*p* = .12, Cohen’s d = 0.71). These findings indicate that outdoor sports education was associated with lower fear of failure among university students.

**Table 5:** ANCOVA results for group differences in fear of failure

		Pre-test	Post-test	F	P	η²p
Fear of experiencing embarrassment	Intervention	4.40 ± 0.53	3.32 ± 0.20	8.94	0.003	0.12
	Control	4.38 ± 0.37	3.91 ± 0.21			
Fear of devaluation by others	Intervention	4.36 ± 0.51	2.97 ± 0.31	9.12	0.002	0.13
	Control	4.34 ± 0.35	3.58 ± 0.35			
Fear of others losing interest	Intervention	4.42 ± 0.54	3.56 ± 0.25	8.23	0.004	0.11
	Control	4.40 ± 0.38	4.13 ± 0.28			
Fear of disappointing important others	Intervention	4.34 ± 0.50	3.41 ± 0.18	2.08	0.151	0.03
	Control	4.32 ± 0.34	3.50 ± 0.26			
Total Score	Intervention	4.38 ± 0.52	3.32 ± 0.19	8.76	0.003	0.12
	Control	4.36 ± 0.36	3.78 ± 0.23			

### Common method bias test

To mitigate and assess the potential common method bias arising from self-reported measures, this study implemented both procedural controls (e.g., reverse scoring on selected dimensions, anonymous responses) and statistical approaches. Harman’s single-factor test was applied to analyze the data for common method bias. Exploratory factor analysis extracted more than one factor, with the first factor accounting for 29.85% of the total variance—below the critical threshold of 40%. These findings indicate that there was no obvious common method bias in the present data.

### Correlation snalysis among outdoor sports education, employment anxiety, self-management, and fear of failure

Correlation analyses (Table 6) revealed several notable associations. Outdoor sports education correlated strongly with Personal Ability, Emotion Management, Cognition Management, and two fear-of-failure dimensions (Fear of Experiencing Embarrassment and Fear of Devaluation by Others). Employment anxiety was similarly associated with Behavior Management and Emotion Management from the self-management construct, and with the same two fear-of-failure dimensions. Furthermore, fear of failure was significantly correlated with all dimensions of both employment anxiety and self-management, displaying particularly high correlations with Knowledge Application and Behavior Management. Although several subdimensions of self-management showed high intercorrelations (*r* > .70), multicollinearity diagnostics confirmed that these correlations did not adversely affect the stability of the regression coefficients (all VIF < 3.0). After applying the Bonferroni correction for multiple comparisons, the pattern of significant correlations remained largely unchanged, indicating robust associations among the key variables.

**Table 6:** Correlations Among Variables

	1	2	3	4	5	6	7	8	9	10	11	12	13
1.OSE	1												
2.PA	0.508*	1											
3.KA	0.323*	0.637*	1										
4.CR	0.398*	0.543*	0.487*	1									
5.SR	0.290	0.396*	0.463*	0.276	1								
6.BM	0.681*	0.618*	0.589*	0.387*	0.421*	1							
7.EM	0.519*	0.570*	0.636*	0.411*	0.398*	0.698*	1						
8.TM	0.386*	0.352*	0.578*	0.218	0.419*	0.701*	0.470*	1					
9.CM	0.473*	0.477*	0.499*	0.193	0.331*	0.560*	0.558*	0.532*	1				
10.FEE	− 0.572*	− 0.351*	− 0.536*	− 0.336*	− 0.360*	− 0.523*	− 0.337*	− 0.463*	− 0.631*	1			
11.FEV	− 0.596*	− 0.471*	− 0.507*	− 0.387*	− 0.357*	− 0.560*	− 0.471*	− 0.581*	− 0.550*	0.535*	1		
12.FOL	− 0.371*	− 0.510*	− 0.385*	− 0.510*	− 0.301*	− 0.480*	− 0.359*	− 0.373*	− 0.490*	0.761*	0.689*	1	
13.FEI	− 0.460*	− 0.462*	− 0.411*	− 0.343*	− 0.361*	− 0.526*	− 0.433*	− 0.298	− 0.533*	0.566*	0.571*	0.613*	1

Note: *OSE* Outdoor Sports Education, *PA* Personal Ability, *KA* Knowledge Application, *CR* Career Replaceability, *SR *Social Relations, *BM *Behavior Management, *EM *Emotion Management, *TM *Time Management, *CM *Cognition Management, *FEE *Fear of Experiencing Embarrassment, *FDV *Fear of Devaluation by Others, *FOL *Fear of Others Losing Interest, *FDI *Fear of Disappointing Important Others. *p < .001 (Bonferroni-corrected)

### Structural model fit assessment

Prior to testing the structural models, gender was included as a covariate in all analyses to control for its potential confounding effects, given the significant gender differences observed in the preliminary analyses. A direct effect model was first tested, with outdoor sports education as the independent variable and employment anxiety as the dependent variable. The model showed acceptable fit indices: χ²/df = 6.18, CFI = 0.93, TLI = 0.91, RMSEA = 0.08, SRMR = 0.04. Although the χ²/df ratio exceeded the conventional threshold of 5, this is likely attributable to the relatively large sample size (*N* = 307), as the χ² statistic is sensitive to sample size. Given the acceptable values of other fit indices (CFI > 0.90, RMSEA < 0.08), the model was considered to have adequate fit. Results indicated that outdoor sports education was a significant positive predictor of employment anxiety.

Subsequently, a chain mediation model was examined, incorporating fear of failure and self-management as mediators, with gender again included as a covariate. This model demonstrated good fit to the data: χ²/df = 5.91, CFI = 0.96, TLI = 0.93, RMSEA = 0.06, SRMR = 0.05. Although the χ²/df ratio exceeds the conventional threshold of 5, the model is considered acceptable given the sample size sensitivity of the χ² statistic and the satisfactory values of other fit indices (CFI = 0.96, RMSEA = 0.06). The detailed path coefficients and the structural diagram of the chain mediation model are presented in Fig. 2.

To examine the mediating effects, bootstrap analyses with 5,000 resamples and bias-corrected 95% confidence intervals (CIs) were conducted, with gender included as a covariate in all models. All effects reported are standardized coefficients (β), allowing for comparison across pathways. The proportion of the total effect accounted for by each specific effect was calculated as (specific effect / total effect) × 100%. As summarized in Table 7, the direct effect of outdoor sports education (OSE) on employment anxiety (EA) was significant (β = − 0.16, 95% CI [–0.27, − 0.12]), accounting for 28.1% of the total effect. The indirect effect mediated by self-management (SM) was − 0.15 (95% CI [–0.23, − 0.10]), explaining 26.3% of the total effect. The indirect effect via fear of failure (FF) was − 0.19 (95% CI [–0.31, − 0.11]), contributing 33.3%. The chain-mediated effect through both SM and FF was − 0.07 (95% CI [–0.18, − 0.02]), representing 12.3% of the total effect. In summary, both SM and FF independently mediated the relationship between OSE and EA, and they also operated sequentially as a chain mediator, jointly explaining a substantial portion of the total effect.

**Table 7:** Results of Bootstrap Analyses

Mediating path	Effect	LLCI	ULCI	Proportion of total effect
OSE→EA	−0.16	−0.27	−0.12	28.1%
OSE→SM→EA	−0.15	−0.23	−0.10	26.3%
OSE→FF→EA	−0.19	−0.31	−0.11	33.3%
OSE→SM→FF→EA	−0.07	−0.18	−0.02	12.3%
Total effect	−0.57	−0.71	−0.39	100%

Note: *OSE *Outdoor Sports Education, *EA *Employment Anxiety, *SM *Self-Management, *FF *Fear of Failure. All effects are standardized coefficients (β). *LLCI *lower limit of bias-corrected 95% confidence interval, *ULCI *upper limit of bias-corrected 95% confidence interval

## Discussion

### Associations between outdoor sport education and employment anxiety

University students navigate a critical life transition, facing multifaceted stressors including leaving home, establishing independence, assuming adult responsibilities, and managing academic demands. Research indicates that a substantial proportion of students report heightened perceived stress, whereby environmental demands are appraised as exceeding coping resources, often resulting in persistent negative emotions such as anxiety and depression [23]. Chronic psychological stress is not only closely linked to academic performance, campus involvement, and attrition rates but may also constitute a significant long-term risk to mental health [24]. The present study suggests that university students commonly experience employment anxiety, along with challenges related to fear of failure and self-management, with female students reporting comparatively higher levels of anxiety and fear of failure.

In this context, outdoor sports education may represent an an innovative intervention with demonstrated potential to alleviate employment anxiety and enhance psychological adaptation. Our findings indicate that outdoor sports education was associated with reduced employment anxiety, and that this association may be explained in part by improvements in fear of failure and self-management, underscoring its distinctive educational value. This aligns with research suggesting that outdoor environments facilitate direct observation, experiential learning, and contextualized cognition, promoting attentional restoration, reducing mental fatigue, and fostering positive affect [25].

Regarding employment anxiety, outdoor sports not only provide a physical outlet for stress reduction but also leverage the restorative qualities of natural settings to regulate emotion and build psychological resilience. Goal-setting, overcoming challenges, and achieving success in outdoor activities can strengthen students’ self-efficacy, while the inherent uncertainty and variability of natural environments offer practical contexts for developing adaptive coping and an optimistic outlook [26]. Through such mechanisms, outdoor sports education was associated with enhanced psychological resilience and self-efficacy, equipping students with a more positive mindset to navigate employment anxiety, fear of failure, and related concerns. Supporting this, students with positive psychological dispositions are more likely to employ problem-focused coping strategies, which are in turn associated with lower levels of employment anxiety [27].

In summary, outdoor sports education represents not merely a physical intervention but a multidimensional educational model that integrates psychological, behavioral, and environmental components. By enhancing self-management competence, modulating fear of failure, offering restorative natural exposure, and accumulating positive psychological resources, it may serve as a useful approach to mitigating employment anxiety among university students.

### The mediating role of self-management in the association between outdoor sport education and employment anxiety

In the context of today’s highly competitive labor market, employment anxiety has emerged as a prominent issue adversely affecting the mental health and career development of university students [28]. Through a systematic investigation of the relationships between outdoor sports education, self-management, and employment anxiety, this study found that self-management capacity may represent a key mechanism through which outdoor sports education is associated with psychological benefits. Self-management is defined as the ability to integrate and regulate personal resources across cognitive, emotional, behavioral, and temporal domains [29]. Findings indicate that outdoor sports education does not directly reduce employment anxiety; rather, it exerts a psychologically protective influence by substantially enhancing students’ self-management capabilities [30].This mechanism is consistent with the Conservation of Resources Theory, whereby outdoor sports education facilitates the accumulation and optimization of personal psychological resources—specifically self-management competencies—thereby improving students’ efficiency in mobilizing and allocating these resources under employment-related stress [31].

The distinctive value of outdoor sports education resides in its structured, experiential approach to systematically developing self-management skills. At the behavioral level, goal-setting and task execution in outdoor activities help students establish coherent action sequences from planning to implementation [32, 33]. In emotional management, navigating challenges in natural settings fosters emotional awareness and regulatory skills. Regarding time management, training in pacing and activity rhythm enhances the effective allocation of temporal resources [34]. In the cognitive domain, adapting to complex environmental demands strengthens cognitive flexibility and evaluative capacities. Together, these four dimensions constitute an integrated self-regulatory system that is critical for addressing employment-related challenges.

Notably, the self-management competencies cultivated in outdoor settings demonstrate strong real-world transferability. Skills such as goal setting, emotion regulation, and time planning acquired in natural environments can be directly applied to self-management strategies during the job-search process [35]. This transfer suggests that outdoor sports education may offer benefits beyond conventional physical exercise, potentially serving as a useful approach to fostering career adaptability. Educational practice further suggests that explicitly embedding self-management training within outdoor curricula, designing progressively challenging tasks, and providing timely behavioral feedback can optimize the psychological benefits of such programs [36].

In summary, outdoor sports education equips university students with essential psychological and behavioral resources to cope with employment pressure by systematically enhancing their self-management abilities. Self-management appears to be not only directly associated with lower anxiety but also linked to it through multiple protective pathways. These findings imply that interventions aimed at reducing employment anxiety should extend beyond improving external labor-market conditions and actively promote the cultivation of intrinsic self-management competencies through experiential learning approaches.

### The mediating role of fear of failure in the association between outdoor sport education and employment anxiety

Fear of failure is conceptualized as a pattern of excessive concern among university students regarding potential negative evaluations, self-doubt, and career setbacks during the job-search process, predominantly reflected in two dimensions: fear of embarrassing experiences and fear of devaluation by others [37]. The present study found that fear of failure appeared to mediate the relationship between outdoor sports education and employment anxiety, functioning as a pivotal psychological factor in both the development and mitigation of such anxiety. Outdoor sports offer structured yet challenging scenarios that allow participants to engage in “simulated failure” within a contained and supportive setting. Through such graduated exposure, students’ sensitivity to failure is diminished, and their cognitive appraisal of setbacks is constructively reframed [38]. Consistent with this, our results indicated that students who participated in outdoor sports education scored significantly lower on the dimensions of fear of embarrassing experiences and fear of devaluation by others. In group-based outdoor activities, failure is reconceptualized not as a definitive judgment but as an integral part of the learning process. Observing peers’ responses to challenges helps students internalize a growth-oriented perspective, wherein failure is viewed as a normative and instructive phase of development, thereby attenuating catastrophic interpretations of setbacks [39, 40]. Moreover, repeated experiences of overcoming outdoor challenges bolster participants’ self-efficacy, a sense of competence that can be transferred to employment-related contexts, empowering students to confront the uncertainties of the job market with increased resilience [41].

The dynamic and unpredictable nature of outdoor environments parallels the volatility of contemporary labor markets, rendering coping strategies developed in such settings particularly transferable [42]. Natural contexts furnish immediate and tangible feedback on action outcomes, enabling students to refine self-assessment skills and counteract fear-amplifying cognitive distortions [43]. Furthermore, the multi-layered support system inherent in group outdoor activities—combining peer encouragement and instructor guidance—serves to buffer the psychological impact of failure experiences [44]. Although not directly tested, the pattern of results suggests a potential interactive relationship between self-management and fear of failure warranting future investigation. Collectively, these findings position fear of failure as a psychological conduit through which outdoor sports education is associated with reduced employment anxiety.

Students with enhanced self-management competencies, as developed through outdoor sports education, are better equipped to contextualize and derive meaning from failure, thereby diminishing its distressing impact [45]. Collectively, these findings position fear of failure as a critical psychological conduit through which outdoor sports education contributes to the reduction of employment anxiety. In summary, by facilitating controlled exposure to failure, fostering cognitive restructuring, and strengthening psychosocial support mechanisms, outdoor sports education was associated with lower university students’ fear of failure, which in turn mediates the alleviation of employment anxiety.

### The chain mediation role of self-management and fear of failure in the associations between outdoor sport education and employment anxiety

This study found that outdoor sports education is associated with employment anxiety not only through direct effects but also via two interrelated psychological pathways: an emotional regulation pathway mediated by fear of failure and a behavioral competency pathway mediated by self-management. Together, these mechanisms constitute an integrated intervention framework. As an emotional mediator, fear of failure operates principally through exposure-desensitization and cognitive restructuring processes [46]. This pathway underscores the foundational importance of emotional regulation in interventions. By engaging students in controlled, challenging outdoor scenarios, the program facilitates repeated exposure to “micro-failures” within a supportive context, thereby reducing fears of embarrassment and social devaluation [47]. Such structured experiences not only alleviate the affective weight associated with anticipated failure but also promote a reappraisal of failure as a constructive component of learning, disrupting the self-perpetuating cycle of failure and anxiety.

Concurrently, self-management acts as a competency-based mediator, fostering the development of multidimensional self-regulatory skills. Outdoor sports education enhances students’ capabilities across four core domains: behavioral control, emotional regulation, time management, and cognitive flexibility [48]. This skill set may help students approach job-search activities with greater organization, regulate emotional responses more effectively, allocate personal resources efficiently, and ultimately build resilience against employment-related stressors [49]. Importantly, the two pathways do not function in isolation but interact sequentially within a chain-mediation model. The sequential pattern observed in the chain mediation model is consistent with the theoretical proposition that competency development may facilitate emotional regulation. Specifically, heightened self-management capacity appears to be associated with lower fear of failure, which in turn relates to reduced employment anxiety. However, as this interpretation is based on cross-sectional data, causal sequencing cannot be definitively established. Future research employing longitudinal designs is needed to test the directional relationships implied by the chain mediation model. These sequential dynamics imply that effective interventions should follow a progressive structure: behavioral competency development should precede and support the enhancement of emotional regulation, which subsequently contributes to anxiety reduction [50].

In summary, outdoor sports education was associated with reduced employment anxiety through a dual-pathway model comprising fear of failure reduction and self-management enhancement. While each pathway was associated with independent effects, they also appeared to operate synergistically within the chain mediation model. These findings suggest that interventions may benefit from an integrated developmental approach—one that cultivates self-management competencies while concurrently addressing fear of failure. However, given the cross-sectional nature of the mediation analyses, these interpretations should be considered preliminary, and future longitudinal research is needed to establish causal relationships.

### Limitations and future prospects

Several limitations should be acknowledged when interpreting the findings of this study. First, all variables were measured using self-report questionnaires, which may introduce common method bias. Although Harman’s single-factor test suggested that common method bias was not a major concern (first factor accounted for 29.85% of variance), this approach has limited sensitivity. Future research should employ more rigorous methods to control for common method bias, such as incorporating a latent method factor in confirmatory factor analysis or using multi-source data collection. Second, while randomization was conducted at the class level, intra-class correlation coefficients (ICCs) were calculated and found to be low (0.02–0.05), justifying the use of single-level analyses. Third, the sample was restricted to third-year undergraduate students from a single university in China, which limits the generalizability of the findings. The narrow age range (18–20 years) and specific educational context may not represent other student populations or cultural settings. Fourth, although effect sizes (η²p) were reported for all main comparisons, the study was not designed to detect small effects. Fifth, the cross-sectional nature of the mediation analyses precludes causal conclusions regarding the directional relationships among variables. Although the chain mediation model was theoretically grounded, alternative model specifications (e.g., reversed pathways) cannot be ruled out. Longitudinal designs with multiple time points are needed to establish temporal precedence and strengthen causal inferences. Finally, while the Bonferroni correction was applied to the correlation analysis to control for Type I error, this conservative approach may increase Type II error; thus, findings should be interpreted with appropriate caution.

## Conclusion

This study provides evidence that outdoor sports education is associated with lower employment anxiety, enhanced self-management competencies, and reduced fear of failure among university students in a Chinese educational context. Self-management and fear of failure were found to mediate the relationship between outdoor sports education and employment anxiety, both independently and sequentially. However, these findings should be interpreted within the boundaries of the study’s limitations, including the reliance on self-report measures, the cross-sectional mediation design, and the specific sample characteristics (third-year students from a single Chinese university). Within these contextual boundaries, the results suggest that interventions incorporating outdoor sports education may offer a useful approach to supporting student psychological well-being. Future research should examine whether these findings generalize to other populations and educational settings, and employ longitudinal designs to establish causal relationships.

## Data Availability

The datasets used and/or analyzed during the current study are available from the corresponding author upon reasonable request.
